# Supplementary figures and images for: Psychological impact of the COVID-19 epidemic among healthcare workers in paediatric intensive care units in China
Source: PLoS One. 2022 May 27;17(5):e0265377. doi: 10.1371/journal.pone.0265377 (PMC9140227; doi:10.1371/journal.pone.0265377)

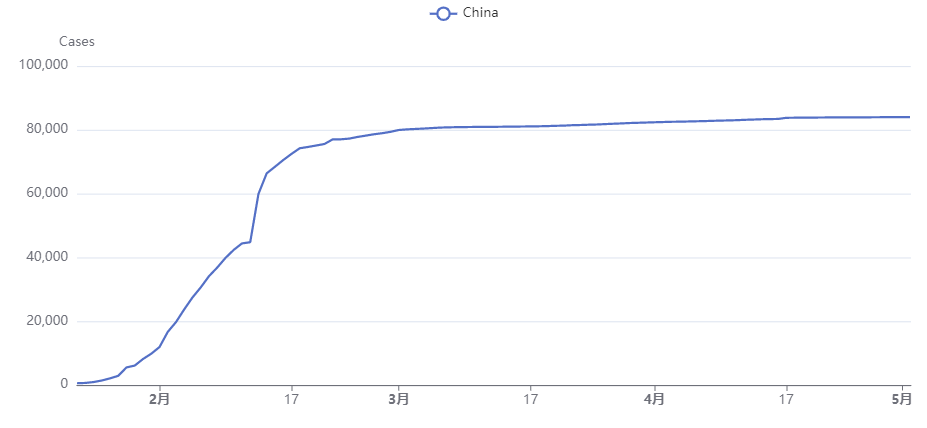

Supplement: S1 Fig — (TIF) [file pone.0265377.s001.tif]

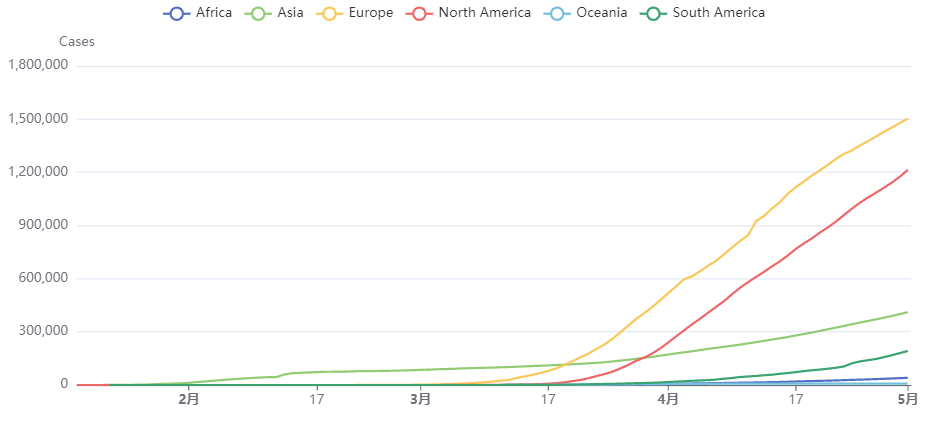

Supplement: S2 Fig — (TIF) [file pone.0265377.s002.tif]

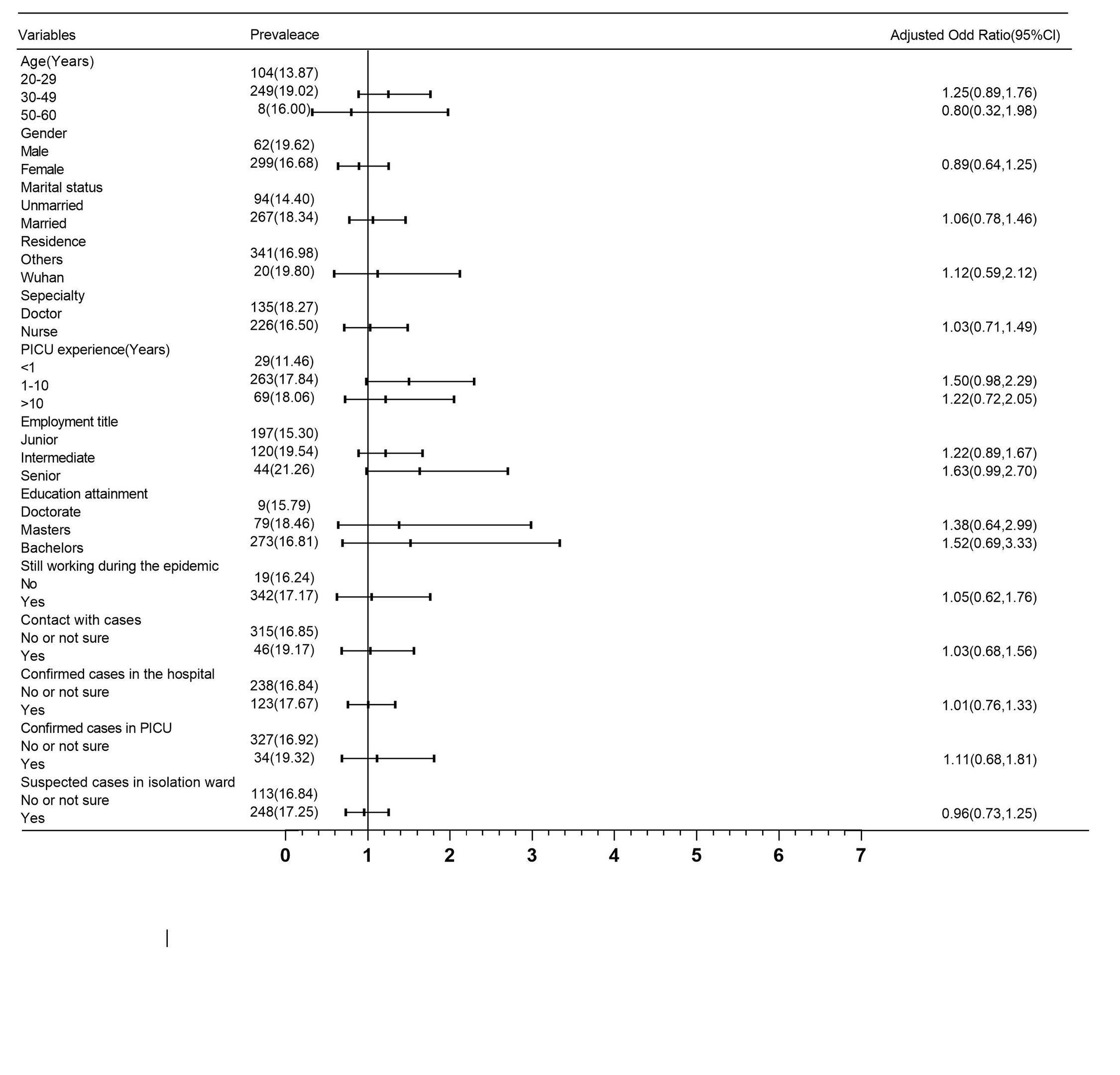

Supplement: S3 Fig — (TIF) [file pone.0265377.s003.tif]
