# Supplementary material for: Psychological impact of the COVID-19 epidemic among healthcare workers in paediatric intensive care units in China
Source: PLoS One. 2022 May 27;17(5):e0265377. doi: 10.1371/journal.pone.0265377 (PMC9140227; doi:10.1371/journal.pone.0265377)
Supplement: S1 Table — (DOCX) [file pone.0265377.s004.docx]

| Symptoms | Post-traumatic distress | Depression | Anxiety | Stress |
| --- | --- | --- | --- | --- |
| Normal | 1139(54.01) | 1272(60.31) | 1340(63.54) | 1748(82.88) |
| Mild | 476(22.57) | 284(13.47) | 295(13.99) | 191(9.06) |
| Moderate | 137(6.50) | 455(21.57) | 320 (15.17) | 102(4.84) |
| Severe | 357(16.93) | 48(2.28) | 92(4.36) | 47(2.23) |
| Extremely severe | - | 50(2.37) | 62(2.94) | 21(1.00) |

Supplementary Table 1. Percentage of participants with mild to extremely severe PTS, depression, anxiety and stress. PTS: Posttraumatic stress.
