# Supplementary material for: Psychological impact of the COVID-19 epidemic among healthcare workers in paediatric intensive care units in China
Source: PLoS One. 2022 May 27;17(5):e0265377. doi: 10.1371/journal.pone.0265377 (PMC9140227; doi:10.1371/journal.pone.0265377)
Supplement: S2 Table — (DOCX) [file pone.0265377.s005.docx]

|  | IES-R | | Depression | | Anxiety | | Stress | |
| --- | --- | --- | --- | --- | --- | --- | --- | --- |
|  | Χ^2^ | P value | Χ^2^ | P value | Χ^2^ | P value | Χ^2^ | P value |
| Age |  |  |  |  |  |  |  |  |
| 20-29 v.s. 30-49 | 32.83 | **<0.001** | 9.89 | **<0.01** | 0.73 | 0.39 | 8.92 | **<0.01** |
| 20-29 v.s. 50-60 | 16.34 | **<0.001** | 0.47 | 0.49 | 1.15 | 0.28 | 0.18 | 0.67 |
| 30-49 v.s. 50-60 | 4.77 | **0.03** | 0.10 | 0.75 | 1.81 | 0.18 | 0.29 | 0.59 |
| PICU experience |  |  |  |  |  |  |  |  |
| <1 v.s. 1-10 | 11.80 | **<0.01** | 4.91 | **0.03** | 3.31 | 0.07 | 6.28 | **0.01** |
| <1 v.s. >10 | 28.26 | **<0.001** | 8.91 | **<0.01** | 4.43 | **0.04** | 5.08 | **0.02** |
| 1-10 v.s. >10 | 11.94 | **<0.01** | 2.53 | 0.11 | 0.65 | 0.42 | 0.01 | 0.92 |
| Employment title |  |  |  |  |  |  |  |  |
| Junior v.s. Intermediate | 8.01 | **<0.01** | 7.41 | **<0.01** | 0.23 | 0.63 | 0.28 | 0.59 |
| Junior v.s. Senior | 23.76 | **<0.001** | 12.44 | **<0.001** | 0.01 | 0.94 | 4.69 | **0.03** |
| Intermediate v.s. Senior | 7.76 | **<0.01** | 0.77 | 0.38 | 0.46 | 0.50 | 5.41 | **0.02** |

Supplementary Table 2. T-tests results for psychological states differences between different age groups, PICU experience and employment title.
