# Supplementary material for: Psychological impact of the COVID-19 epidemic among healthcare workers in paediatric intensive care units in China
Source: PLoS One. 2022 May 27;17(5):e0265377. doi: 10.1371/journal.pone.0265377 (PMC9140227; doi:10.1371/journal.pone.0265377)
Supplement: Attachment — Submitted filename: Review_PONE-D-21-13813_Plos one.docx [file pone.0265377.s007.docx]

**Reviewer#**

**Comments to the Author**

**Summary:**

The study aimed to explore the psychological impact of the COVID-19 outbreak on
Medical workers and analyses their independent risk factors. The data were based on 2123 medical workers, and the findings show that during the COVID-19 epidemic, the mental health status of
PICU medical workers were affected to varying degrees in terms of stress, depression
and anxiety. Marital status, job title, education level, working status and degree of
contact with COVID-19 were independent risk factors.

However, there still were some notable deficiencies in the article.

**Abstract**
1. **When writing about PICUs for the first time, the author should give its full name or meaning?**

**Introduction**

**2.Page 4, lines 71-72. Please briefly revisit and summarize the research gaps. There is no link between the study aim and the previous sentence statement.**

**Method:**

**3. Page 4, Line 87-90 The reason for not taking consent is unclear from the sentence.**

**4. Are there any inclusion and exclusion criteria for sample size?**

**5. What is the value of Cronbach's α? How does the author has collected the data of Cronbach's α values in the present study?**

**Results:**

**6. Page 6, Line 127 to 136 It is unclear what percentage and number mentioned are for what? The author needs to rephrase the sentence.**

**7. Please provide legends in the table for better understanding.**

**Discussion**

**8.      There is no real discussion with the data; it is very descriptive. The discussion should not simply discuss the similarities and differences with previous studies.**

**9. Author need to provided citations whenever they are comparing their finding with prior research. E.g. page 15, line 199 to 202 and 207 to 213**

**10. Page 16, Line 227 to 229 what are these scores, and how is it related to social responsibilities?**

**11. It is hard to understand the discussion point made by the author. I think the author needs to make it understandable to readers with proper citation wherever required.**

**12. Author has just mentioned about limitation of the study; please provide strength**

**13. Author has mentioned limitations in conclusion. Authors are suggested to phrase a subheading name "Strengths and limitations" after discussion, and within this subsection, put the strengths first, followed by the study's limitations.**

**14. I see the English writing of this paper can be improved, not only grammar but the way how the statements were phrased were not concise enough.**
