# Supplementary material for: Psychological impact of the COVID-19 epidemic among healthcare workers in paediatric intensive care units in China
Source: PLoS One. 2022 May 27;17(5):e0265377. doi: 10.1371/journal.pone.0265377 (PMC9140227; doi:10.1371/journal.pone.0265377)
Supplement: Attachment — Submitted filename: Response to Reviewers.docx [file pone.0265377.s008.docx]

Dear Editor,

On behalf of my-authors, we thank you very much for giving us an opportunity to revise our manuscript. We appreciate editor and reviewers very much for their positive and constructive comments and suggestions on our manuscript entitled “Psychological Impact of the COVID-19 Epidemic among Healthcare Workers in paediatric intensive care units in China” (manuscript PONE-D-21-13813). To address the critiques of the reviewers, we revised our manuscript according to their comments. Attached please find the revised version (All changes are marked as red color), which we would like to submit for you kind consideration. We would like to express our great appreciation to you and reviewers for comments on our paper.

Looking forward to hearing from you. Thank you and best regards.

Yours sincerely,

Correspondence to:

Yue Zhang

18375763857@163.com

Abstract

1. When writing about PICUs for the first time, the author should give its full name or meaning?

A: We have given its full name.

Introduction

2.Page 4, lines 71-72. Please briefly revisit and summarize the research gaps. There is no link between the study aim and the previous sentence statement.

A: At present, research on the psychological effects of PICU HCWs is very limited. We have re-organized and explained the reasons for choosing PICU HCWs as the research object.

Method:

3. Page 4, Line 87-90 The reason for not taking consent is unclear from the sentence.

A: All participants voluntarily responded to the survey anonymously and provided informed consent online before the survey.

4. Are there any inclusion and exclusion criteria for sample size?

A: Since the questionnaire was completed voluntarily, there are no inclusion and exclusion criteria.

5. What is the value of Cronbach's α? How does the author has collected the data of Cronbach's α values in the present study?

A: The scale we chose is a mature Liszt scale, so there is no calculation about reliability and validity.

Results:

6. Page 6, Line 127 to 136 It is unclear what percentage and number mentioned are for what? The author needs to rephrase the sentence.

A: We have rephrased the sentence.

7. Please provide legends in the table for better understanding.

A: We have provided legends in the table.

Discussion

8. There is no real discussion with the data; it is very descriptive. The discussion should not simply discuss the similarities and differences with previous studies.

9. Author need to provided citations whenever they are comparing their finding with prior research. E.g. page 15, line 199 to 202 and 207 to 213

10. Page 16, Line 227 to 229 what are these scores, and how is it related to social responsibilities?

11. It is hard to understand the discussion point made by the author. I think the author needs to make it understandable to readers with proper citation wherever required.

A: On the basis of past research, we have re-discussed and analyzed the data.

12. Author has just mentioned about limitation of the study; please provide strength

13. Author has mentioned limitations in conclusion. Authors are suggested to phrase a subheading name "Strengths and limitations" after discussion, and within this subsection, put the strengths first, followed by the study's limitations.

A: We have phrased a subheading name "Strengths and limitations" after discussion.

14. I see the English writing of this paper can be improved, not only grammar but the way how the statements were phrased were not concise enough.

A: We have submitted our manuscript to “AJE” for language polishing.
