# Supplementary material for: Psychological impact of the COVID-19 epidemic among healthcare workers in paediatric intensive care units in China
Source: PLoS One. 2022 May 27;17(5):e0265377. doi: 10.1371/journal.pone.0265377 (PMC9140227; doi:10.1371/journal.pone.0265377)
Supplement: Attachment — Submitted filename: Response to Reviewers.docx [file pone.0265377.s009.docx]

Dear Reviewer,

On behalf of my-authors, we thank you very much for giving us an opportunity to revise our manuscript. We appreciate you very much for positive and constructive comments and suggestions on our manuscript entitled “Psychological Impact of the COVID-19 Epidemic among Healthcare Workers in paediatric intensive care units in China” (manuscript PONE-D-21-13813). To address the critiques, we revised our manuscript according to comments. We have submitted our manuscript to “AJE” for premium editing again and will respond to each point. Attached please find the revised version (All changes are marked as red color), which we would like to submit for you kind consideration. We would like to express our great appreciation to you for comments on our paper.

Looking forward to hearing from you. Thank you and best regards.

Yours sincerely,

Correspondence to:

Yue Zhang

18375763857@163.com

1. Please carefully check the citation required in the manuscript for eg. Line no. 424-427 page 20 and Line 434- 434

A: We have inserted the citation in the appropriate position of the article.

2. What does author mean by the positive rate of HCWs seems to be lower in Line no. 438 page 20?

A: It refers to the stress level of HCWS, which has been changed.

3. Line no. page 20 What does author mean by following sentence. “However, the greater difference between married and unmarried HCWs is children.” Kindly rephrase it to make it meaningful.

A: Based on the traditional concept of "marrying and giving birth to children" in Chinese families, it means that the greater difference between married and unmarried HCWs is children.

4. Further, I would like to suggest author to change the first paragraph of discussion, its similar to the introduction.

A: After discussion, we also agree with your views. Without affecting our discussion, the first paragraph of discussion has been deleted.
